# Live Imaging of Cell Motility and Actin Cytoskeleton of Individual Neurons and Neural Crest Cells in Zebrafish Embryos

**DOI:** 10.3791/1726

**Published:** 2010-02-03

**Authors:** Erica Andersen, Namrata Asuri, Matthew Clay, Mary Halloran

**Affiliations:** Genetics Training Program, University of Wisconsin-Madison; Department of Anatomy, University of Wisconsin-Madison; Department of Zoology, University of Wisconsin-Madison; Cell and Molecular Biology Training Program, University of Wisconsin-Madison

## Abstract

The zebrafish is an ideal model for imaging cell behaviors during development *in vivo*. Zebrafish embryos are externally fertilized and thus easily accessible at all stages of development. Moreover, their optical clarity allows high resolution imaging of cell and molecular dynamics in the natural environment of the intact embryo. We are using a live imaging approach to analyze cell behaviors during neural crest cell migration and the outgrowth and guidance of neuronal axons.

Live imaging is particularly useful for understanding mechanisms that regulate cell motility processes. To visualize details of cell motility, such as protrusive activity and molecular dynamics, it is advantageous to label individual cells. In zebrafish, plasmid DNA injection yields a transient mosaic expression pattern and offers distinct benefits over other cell labeling methods. For example, transgenic lines often label entire cell populations and thus may obscure visualization of the fine protrusions (or changes in molecular distribution) in a single cell. In addition, injection of DNA at the one-cell stage is less invasive and more precise than dye injections at later stages.

Here we describe a method for labeling individual developing neurons or neural crest cells and imaging their behavior *in vivo*. We inject plasmid DNA into 1-cell stage embryos, which results in mosaic transgene expression. The vectors contain cell-specific promoters that drive expression of a gene of interest in a subset of sensory neurons or neural crest cells. We provide examples of cells labeled with membrane targeted GFP or with a biosensor probe that allows visualization of F-actin in living cells^1^.

Erica Andersen, Namrata Asuri, and Matthew Clay contributed equally to this work.

**Figure:**
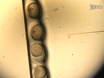


## Protocol

### 1. Assembly of injection slides and imaging slides

#### Injection slides:

Prepare Sylgard silicone elastomer according to manufacturer's instructions.
Use the Sylgard to bond three standard glass microscope slides together by stacking one slide on top of the intersection of the other two slides, which are arranged side-by-side at the long edges. The top slide creates a right-angled corner or "wall" between the top and bottom slides.
Let Sylgard set overnight. These slides are reusable.

#### Imaging slide:

We use stainless steel rectangles cut to the same size of a standard glass microscope slide (75mm X 25mm X 1mm) so the chamber fits the slide holder on the microscope stage. A 1.5 cm hole is cut in the center of the rectangle.
Affix a 22 mm^2^ coverslip to the metal rectangle with Sylgard. Use just enough Sylgard to thoroughly seal the coverslip. To image on an inverted microscope, embryos will be mounted on this coverslip and imaged from the bottom.
Let Sylgard set overnight. These slides are reusable and cleaned with 70% ethanol between uses.

### 2. Preparation of DNA constructs

To express a transgene in a tissue-specific manner, the gene of interest is cloned behind a cell-specific promoter. We use a* cis-*regulatory element of the zebrafish* neurogenin1* gene (*-3.1ngn1)*^2^ or the* sox10 *gene* (-4.9sox10)*^3^ to drive expression in sensory neurons or neural crest cells, respectively. To visualize cell and membrane dynamics, we express cytoplasmic or membrane-localized fluorophores. To image F-actin distribution, we express DNA encoding the calponin homology domain of utrophin fused to mCherry (UtrCH-mCherry). The UtrCH-mCherry biosensor probe allows visualization of F-actin without interfering with actin dynamics^1^. We generate these DNA constructs using the Invitrogen Gateway recombination-based cloning strategy^4^ and vectors provided in the zebrafish Tol2kit^5^.

Generate expression vector(s) using Multisite-Gateway Technology and vectors from the zebrafish Tol2kit^5^. Plasmids contain the Tol2 backbone from pT2KXIGDin.
Purify plasmid DNA with QIAfilter Plasmid Midi Prep kit (QIAGEN Inc.) and elute in sterile water. DNA does not need to be linearized for injection.

### 3. Injection of DNA constructs

Dilute the DNA to a final concentration of 10-50 μg/ml DNA in 0.2% phenol red (for visualization during injection) in water.
Fill and calibrate needle: Back-fill a pulled capillary needle with approximately 1 μl DNA solution and insert it into the needle holder of the injection apparatus (we use a Picospritzer). Using a fine forceps, break the tip off the needle such that the tip opening is approximately 10 mm in diameter. Measure the diameter of the droplet in mineral oil with an ocular micrometer. Adjust pressure duration setting to get a droplet of approximately 1 nl volume.
Collect 1-cell stage embryos in E3 embryo medium (5mM NaCl, 0.17mM KCl, 0.33mM CaCl_2_, 0.33mM MgSO_4_*7H_2_O, pH 7.0).
Transfer approximately 30-60 embryos to the injection slide and position the embryos in the right angle corner formed between the top and bottom slides. Remove most of the E3 so that the embryos are held to the slide by surface tension.
Use a bent dissecting pin to rotate embryos so the cell is against the wall of the injection slide.
Use a micromanipulator to guide the needle through the embryo chorion, through the yolk and into the single cell of the embryo. Inject 0.5-1 nl DNA solution into the cell.
Transfer the embryos into Petri dishes in E3 and incubate at 28.5°C. If necessary, development can be slowed by incubating embryos at lower temperature.

### 4. Mounting embryos for imaging

Remove the chorions from the embryos with fine forceps about an hour before embryos reach the desired age for imaging (approximately 14-17 hours post-fertilization for our purposes).
Select embryos with labeled cells using a dissecting microscope equipped with fluorescence. We use a 4X objective (40X magnification) on a Nikon AZ100 scope because of its combination of long working distance and high magnification.
Place an embryo into a microfuge tube with approximately 50 μl E3 containing 0.2% Tricaine anesthetic (10X concentration).
Add 500 μl 1% low melting point agarose in E3 with 10 mM HEPES (kept at 42°C), diluting the Tricaine to a final concentration of 0.02%.
Immediately transfer the embryo in an agarose drop to the coverslip of the imaging slide using a wide bore glass pipette.
Before the agarose hardens, position the embryo so the region with labeled cells is facing down against the bottom coverslip (dorsal down for neural crest cells and dorsolateral down for spinal sensory neurons).
Assemble imaging chamber: Coat one side of a plastic ring (2 cm outer diameter, 3 mm high) with silicone vacuum grease and affix to the metal imaging slide. Coat the other side of the ring with grease.
Fill the chamber with E3 containing 10 mM HEPES and 0.02% Tricaine. Seal the chamber top by affixing a 22 mm^2^ coverslip to the top greased surface of the ring.

### 5. 4D imaging of cell behaviors on Olympus FV1000 confocal with IX81 microscope

The details of image acquisition will depend on the specifics of your microscope system. Here we describe the time-lapse acquisition process for the Olympus FV1000 confocal microscope. See previous JoVE protocol for time-lapse acquisition with the Zeiss LSM 510 confocal system^6^.

Place the imaging chamber into the slide holder on the microscope stage and focus on the embryo using a 10X or 20X objective under transmitted light.
Switch to a 60X objective (N.A. of 1.2 or higher) and focus on labeled cell with epifluorescence. We use either an oil immersion (UPLSAPO 60xO N.A. 1.35) or water immersion (UPLSAPO 60xW N.A. 1.20) lens.
In the FV software (Ver 1.7c), click on XY repeat to begin laser scanning.
Adjust acquisition settings and image acquisition controls (laser output, scan speed, HV, gain, and offset levels) to optimize signal while keeping laser power at a minimum (25% or lower) to prevent tissue damage and reduce photobleaching.
To acquire a Z-series, set the upper and lower limits of the z-stack, along with step size. Make sure the Depth icon is engaged so that the XY Sweep icon has XY and Z highlighted. Start acquisition by clicking the XYZ Sweep icon.
To acquire a time-lapse series, set the time interval and number of time points under the TimeScan heading in the Acquisition Settings window. Click the Time Icon in the Image Acquisition Control Window so that the XY Sweep icon has XY(Z) and T highlighted. Start acquisition by clicking the XY(Z)T Sweep icon.
Alternatively, the TimeController can be used to set up a time-lapse. This programming is useful when generating large data sets, as it does not exceed memory allocation. Under Device, open TimeController window. Create a new Imaging Task. The image parameter window will open. Click the FV Status button to update acquisition settings. Make sure Save and Delete are checked under the Terminate heading. Set output file name and click OK to save settings and close the window. Use the Loop function to set the desired time interval and number of time points. Click Ready and Start to begin acquisition. The Pause and Z-shift functions can be used to refocus while imaging.

### 6. Representative Results

The figures and movies depict examples of sensory neurons and neural crest cells labeled with membrane-targeted fluorophores or the actin biosensor probe.


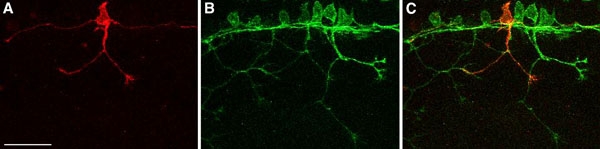
 **Figure 1. **Confocal images (z-projections) of a *Tg(ngn1:gfp-caax)* transgenic embryo injected with 25 pg *ngn1:mCherry-caax* DNA. The mCherry-CAAX labels one neuron red **(A)** in a background with all Rohon-Beard sensory neurons labeled green **(B)**. **C)** Merged image of both green and red channels. Scale bar = 50 μm.


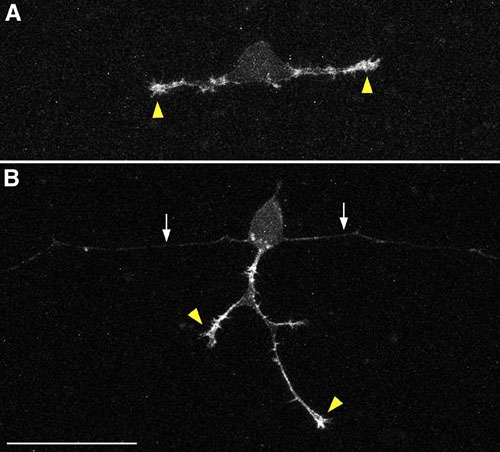
 **Figure 2. **Confocal z-projections of neurons in embryos injected with ~20 pg* ngn1:mCherry-UtrCH* DNA. **A)** Rohon-Beard sensory neuron in 16.5 hpf embryo. F-actin signal is strong in growth cones (arrowheads) and in protrusions along newly formed axons. **B)** Neuron in 20 hpf embryo showing strong F-actin signal in growth cones of branched peripheral axon (arrowheads) and weaker signal in more mature central axons (arrows). Scale bar = 50 μm.

## Discussion

The optimal concentration of injected DNA will vary depending on size and strength of the promoter construct and should be determined empirically. Injection of too much DNA can lead to unhealthy embryos with extensive cell death, while too little will result in a very small proportion of injected embryos expressing the transgene. The DNA expression level correlates with the strength of the fluorophore signal, which varies from cell to cell. While sorting embryos under epifluorescence, exclude those with extremely high levels of expression. Labeled cells that appear very bright usually also show obvious signs of toxicity, such as abnormal cell morphology, mislocalization of tagged molecules, and cell death. A typical DNA injection yields 5-10% of embryos that are suitable for imaging.

The F-actin biosensor can function in a dominant negative fashion when expressed at high levels. If promoter-driven expression is problematic, the biosensor can be expressed ubiquitously by injecting mRNA. The advantage of mRNA injection is that the expression levels can be controlled by adjusting the concentration of mRNA. Individual cells in these embryos are labeled by co-injection of a DNA construct containing a cell-specific promoter driving expression of a membrane-localized fluorophore. We have used this approach to image F-actin in neural crest cells^7^.

We often carry out the single-cell labeling technique in transgenic expression lines. For example, injecting a *-3.1ngn1:mcherry* plasmid into embryos of the *Tg*(*-3.1ngn1:gfp)* line will label individual sensory neurons red in a background of green neurons. This strategy allows examination of the behavior of an individual cell within the context of the entire cell population.

We focus here on imaging neural and neural crest cell behaviors and actin distribution. However, the general method of tissue specific mosaic cell labeling by DNA plasmid injection can be expanded for use with fluorescent fusion proteins as well as other genetically encoded biosensor probes. Combining these techniques can allow investigators to visualize subcellular localization of specific molecules as well as signaling mechanisms* in vivo*.
